# Comparison of the open kinetic chain and closed kinetic chain strengthening exercises on pain perception and lower limb biomechanics of patients with mild knee osteoarthritis: a randomized controlled trial protocol

**DOI:** 10.1186/s13063-022-06153-8

**Published:** 2022-04-15

**Authors:** Wei Hui Ng, Nazatul Izzati Jamaludin, Farhah Nadhirah Aiman Sahabuddin, Shaifuzain Ab Rahman, Amran Ahmed Shokri, Shazlin Shaharudin

**Affiliations:** 1 School of Health Sciences, Universiti Sains Malaysia, 16150 Kota Bharu, Kelantan Malaysia; 2 Klinik Kesihatan Putrajaya Presint 9, Pejabat Kesihatan Putrajaya, 62300 Putrajaya, Wilayah Persekutuan Putrajaya Malaysia; 3 School of Medical Sciences, Universiti Sains Malaysia, 16150 Kota Bharu, Kelantan Malaysia

**Keywords:** Home-based exercise program, Knee osteoarthritis, Physiotherapy

## Abstract

**Background:**

Clinical recommendations suggest exercises as the main treatment modality for patients with knee osteoarthritis (OA). This study aimed to compare the effects of two different exercise interventions, i.e., open kinetic chain (OKC) and closed kinetic chain (CKC) exercises, on the pain and lower limb biomechanics of patients with mild knee OA.

**Method:**

A total of 66 individuals with painful early knee OA, aged 50 years and above, with body mass index (BMI) between 18.9kg/m^2^ and 29.9 kg/m^2^ in Kelantan, Malaysia, will be recruited in this study. Participants will be randomly allocated into three different groups, either the OKC, CKC, or control groups. All three groups will attend an individual session with a physiotherapist. The participants in the OKC and CKC groups will perform the exercises three times weekly for 8 weeks at their home. The control group will receive education about clinical manifestations, risk factors, diagnosis, treatment, and nursing care for knee via printed materials. The primary outcomes include self-reported pain scores (visual analog scale), disability scores (Western Ontario and McMaster Universities Arthritis Index), and quality of life scores (Osteoarthritis Knee and Hip Quality of Life). Secondary outcomes include lower limb biomechanics during gait and sit-to-stand as well as isokinetic knee strength. The outcomes will be measured before and after the intervention.

**Discussion:**

The present study will compare the effects of two different home-based exercise intervention programs among patients with mild knee OA. The study findings will provide vital information that can be used to design an effective exercise program that aims at delaying the OA progression.

**Trial registration:**

The protocol was registered on 22 December 2020 at ClinicalTrials.gov (registration number: NCT04678609).

## Background

Knee OA commonly affects the elderly and also adults in their 40s [1]. The typical clinical symptoms of knee OA include pain, joint stiffness, muscle weakness, impaired proprioception, and reduced joint motion, all of which can affect patients’ daily activities [2]. With a projection of 250% increase of elderly population aged 65 years and above in Malaysia, Singapore, India, Bangladesh, and the Philippines over the next three decades [3], knee OA will significantly impact the healthcare system [4]. According to the Malaysian Clinical Practice for Osteoarthritis Management, 9.3% of adult Malaysians suffered from knee pain, and more than half of those examined had clinical evidence of OA [5]. Additionally, knee OA patients are predisposed to a higher risk of falls than those without knee OA [6]. Worse still, falls can cast a severe impact on the elderly, leading to severe disability or even death [7]. Hence, the prevention of OA development and progression is vital among the elderly with knee OA.

Exercise intervention recommended by clinical guidelines is a crucial non-pharmacological management for knee OA [8]. Exercise intervention appears to be more effective in the early stage of knee OA before the onset of significant structural damage [9, 10]. Several studies suggested that exercise intervention may improve pain, muscle activity, and strength and functional movement of OA patients [11–15]. Biomechanical factors such as gait velocity and knee adduction moment (KAM) are crucial indicators of OA progression. However, much is not known regarding the effects of exercise on the lower limb biomechanics during gait and sit-to-stand (STS) among mild knee OA patients.

To date, open kinetic chain (OKC) and closed kinetic chain (CKC) exercises have been prescribed for knee OA patients. During OKC exercise, the distal segment of the body parts is not fixed, and it can move freely. Knee extension in the sitting position and straight leg raising (SLR) in the lying position are examples of OKC exercises. On the other hand, during CKC exercise, the distal segment of body parts remains stationary on the ground. Examples of CKC exercises include squat and leg press exercises. Both types of exercises were comparable in terms of their impact on improving pain scale, functional scores, and strength [16]. Moreover, 12 weeks of OKC, CKC, and its combination were found to significantly increase the static and dynamic quadriceps strength and thigh muscle bulk in a study that involved 96 patients with knee OA [17]. However, there is a lack of published studies that evaluate the impact of OKC and CKC on the lower limb biomechanics during gait and STS.

Exercise intervention for OA is usually conducted in the hospital or clinic under the supervision of physiotherapists. However, some patients might face logistics constraints in attending the exercise sessions [18]. To improve the adherence to exercise program, the sessions can be relocated from the hospital to other settings such as at home and in the community [7]. Apart from that, patient education is vital to improve the patients’ understanding of their current condition and to ensure compliance to exercise program and long-term self-management at home [12].

The primary aim of this randomized controlled trial (RCT) is to compare the effects of home-based OKC and CKC exercise intervention programs on pain (visual analog scale), disability scores (Western Ontario and McMaster Universities Arthritis Index), and quality of life scores (Osteoarthritis Knee and Hip Quality of Life) among patients with mild knee OA. Our secondary aim is to compare the effects of OKC and CKC exercises on lower limb biomechanics during walking gait and STS tasks. It is hypothesized that OKC and CKC exercises will result in a significant reduction of pain as well as improved functions and quality of life after 8 weeks of intervention. We also hypothesize a small but clinically relevant change in their biomechanical variables following OKC and CKC intervention. These changes may be significant when compared to the usual care group.

## Method/design

### Trial design

**Fig. 1:**
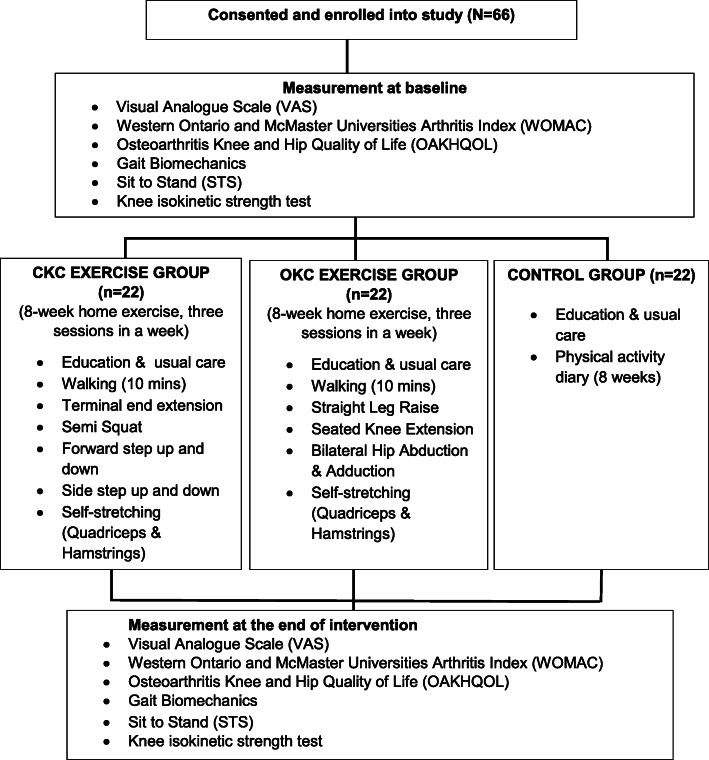
Participant flow through the randomized controlled trial

This study is a randomized controlled trial (RCT) comparing the effects of CKC versus OKC versus the control group in patients with mild knee OA. The effects to be measured include pain, disability, quality of life, and lower limb biomechanics. This protocol will be outlined as per the CONsolidated Standards of Reporting Trials (CONSORT) [19] statement and reported as per Standard Protocol Items: Recommendations for Interventional Trials (SPIRIT) [20]. The flowchart of the study protocol is shown in Fig. 1.

All participants will be assigned randomly to one of the three groups (CKC, OKC, or control groups) by an independent researcher. The allocation numbers will be generated by the study supervisor using a computerized random sequence. The 66 numbers will be inserted in opaque and sealed envelopes until a participant picks an envelope. The participants will then be allocated to the group stated in the envelope. A sealed copy of the randomization code listing will be kept by the study supervisor, and it will not be revealed until the study completion. Additionally, two research team members are designated as outcome assessors for the pre-and post-intervention tests. They will also be blinded to the group allocation and the statistical analyses. Participants will be reminded not to reveal their group allocation to the assessors. The study procedure will follow the Declaration of the Helsinki as approved by the Human Research Ethics Committee at the Universiti Sains Malaysia (JEPeM code: USM/JEPeM/19100645). The protocol has been registered at clinicaltrial.gov (NCT04678609).

The printed information sheet that explains this study will be given to the potential participants together with the consent form. These forms will be produced in a bilingual format in both Malay and English to ensure that all participants understand the research procedure. Participants will give their consent to start the pre-test and intervention sessions by signing the form. The form will also be signed by a witness and the researcher.

### Sample size calculation

The sample size was determined using G*Power statistical software version 3.1.9.2 (Universität Düsseldorf, Germany) based on the primary outcome variables of pain and function based on the visual analog scale (VAS) scores as well as the Western Ontario and McMaster Universities Arthritis (WOMAC) Index respectively. The sample size for this study was determined to detect an effect size of 0.5, based on a previous study [21] with an alpha value of 0.05. Eighteen participants per group will be sufficient to achieve a statistical power of 0.8. By anticipating a dropout rate of 20%, a total of 22 participants per group (total *N* = 66) will be recruited.

### Recruitment of participants

This study will apply purposive sampling for the recruitment of participants. Participants will be included if they:

- Are diagnosed with symptomatic knee OA as defined by the American College of Rheumatology’s Clinical Criteria for Classification and Reporting of knee OA [22–24]
- Are diagnosed with Grade I and II knee OA based on the Kellgren-Lawrence severity scale [25]
- Are female
- Age 50 and older
- Have body mass index (BMI) between 18.5 and 29.9 kg/m^2^
- Have knee pain ≥ two on a VAS

Participants with the following conditions will be excluded:

- Electrical devices implant
- Neurological disorders (i.e., stroke)
- Wheelchair bound
- Significant cognitive impairment
- Present systemic inflammatory arthritis (i.e., gout)
- History of hip or knee arthroplasty
- History of trauma or surgical arthroscopy of either knee in the last 6 months
- Received knee intra-articular injection within the last 3 months
- Under anticoagulant therapy, recent or imminent surgery (within 3 months)
- Involved in any study related to exercise program within the last 6 months

In addition, eligible participants will be considered as drop-outs if they request to withdraw due to any reasons or if they are unable to complete 75% of the intervention program.

### Procedure

Potential participants will first undergo general screening and radiographic screening by the orthopedic medical officer. Disease severity will be assessed using the Kellgren-Lawrence grading system. Pre- and post-intervention assessments will be conducted by the assessors who are blinded to group allocation. These assessments (Fig. 1) will be completed at the sports science laboratory at Universiti Sains Malaysia (USM). All participants will visit USM four times over the 8-week intervention. All participants will also receive the usual care provided by Hospital USM during the 8 weeks of the study, including medication such as pain killers and health education on OA.

### Data collection and management

**Fig. 2:**
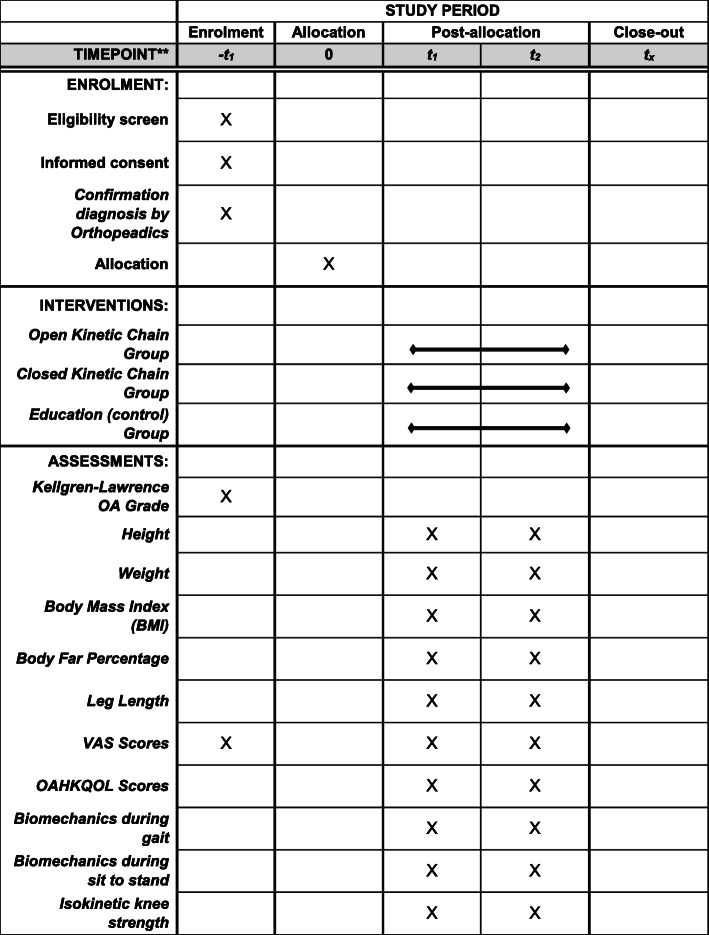
The schedule of enrolment, interventions, and assessments

All the data collection sheets, questionnaires, and printed data of the isokinetic strength test will be stored as hard copy, while the biomechanical data of the gait and STS task will be stored as soft copy in a password-protected computer. Both hard and soft copy data will be stored in a locked room for maintaining security and privacy data. All authors will have access to the final dataset only. The schedule of the enrolment, intervention, and assessment is shown in Fig. 2.

### Interventions

For the CKC and OKC exercise groups, the participants will perform an eight-week individualized home exercise with a minimum of three sessions per week [7]. Each session lasts about 30 min, including 10 min of walking as a warm up session before ending with quadriceps and hamstring stretch as a cool-down session [26–28]. Daily paracetamol (PCM) 3000 mg [24] will be provided by the Hospital USM orthopedic doctor to the participants in all three groups. However, the consumption of PCM is not compulsory, rather depends on each participant's needs. The PCM consumption will be recorded in the exercise diary that is provided. In addition, participants will be contacted through telephone calls and WhatsApp messages three times a week to encourage the participants to adhere to the exercises prescribed [7, 29, 30].

#### The open kinetic chain (OKC) exercise program

**Fig. 3:**
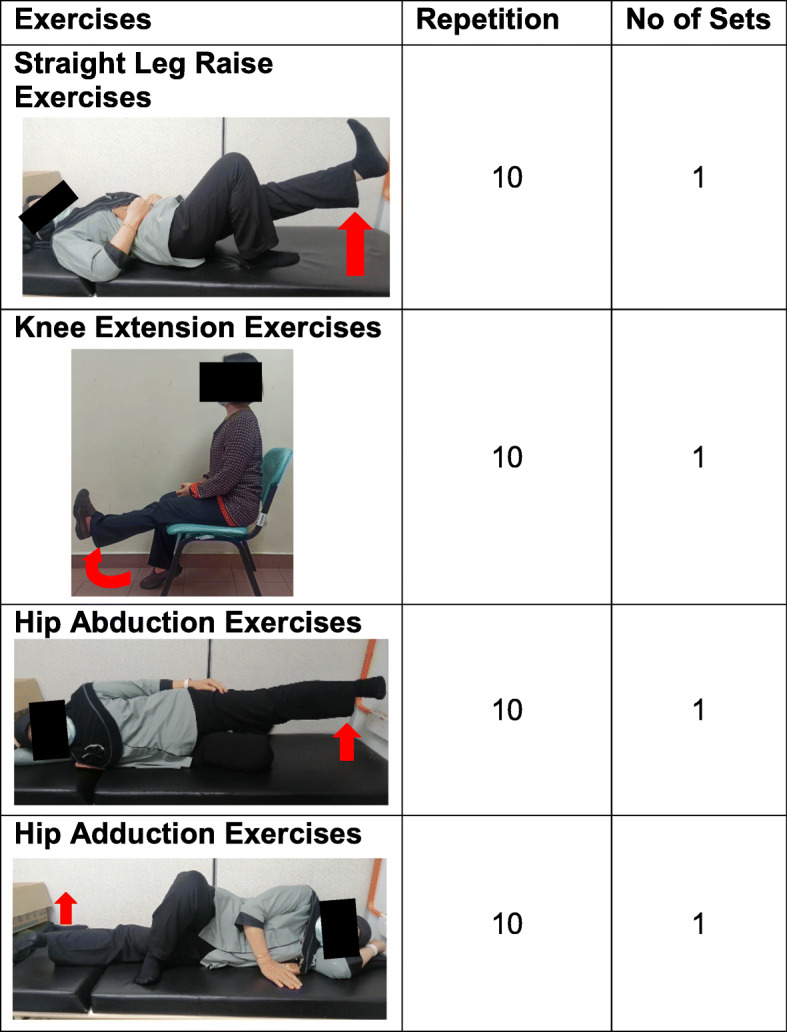
Open kinetic chain exercise program

For the OKC exercise group, participants will perform the following exercises three times a week (Fig. 3):

1) Straight leg raising in lying position;

2) Knee extension in sitting position;

3) Hip abduction in lying position;

4) Hip adduction in lying position.

#### The closed kinetic chain (CKC) exercise program

**Fig. 4:**
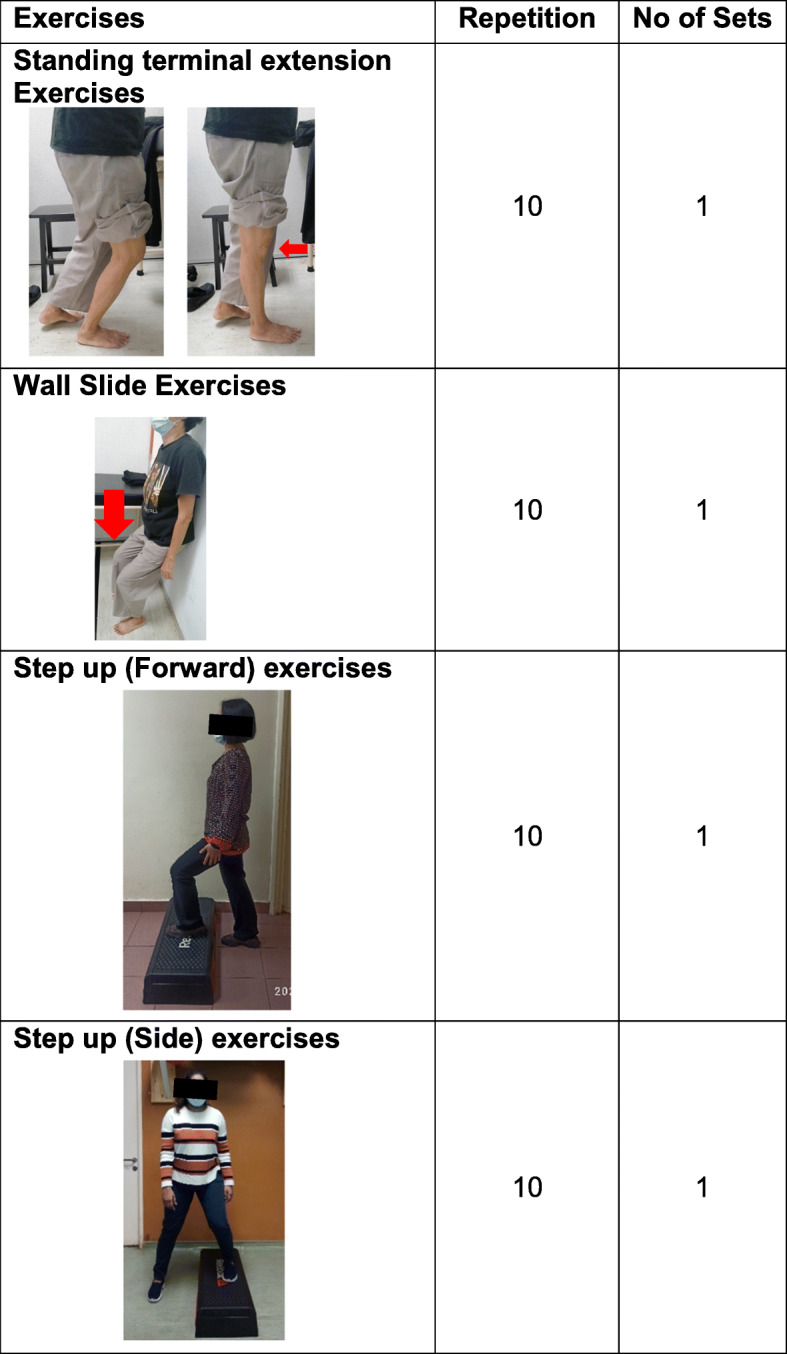
Closed kinetic chain exercise program

For the CKC exercise group, participants will perform the following exercises three times a week (Fig. 4):

1) Terminal end knee extension in standing (maximum 15° of knee flexion) [31];

2) Wall slide (semi squat);

3) Forward step up and down;

4) Side step up and down;

For both OKC and CKC groups, participants will perform all exercises for ten repetitions three times a week, for 8 weeks. Following that, if they consistently obtain less than three on the VAS scale [32], they are encouraged to increase the intensity of the exercise by increasing the number of sets with the same repetitions for each exercise.

#### Control group

All the participants will receive the usual care that includes health education about clinical manifestations, risk factors, diagnosis, treatment, and nursing care for knee OA. However, only the participants in the control group will be given the printed materials of clinical education. Furthermore, the control group will not be exposed to any information related to exercise programs [7]. After completing the trial, the participants in the control group will receive either CKC or OKC exercises depending on their preference.

### Measures

#### Descriptive data

Baseline data about participants’ age, gender, dominant leg, medical history, and medication history will be obtained through the questionnaires. The height (m) and weight (kg) of the participants will be measured with a digital medical scale (Seca 769, Hamburg, Germany). The body fat percentage will be measured by the Tanita Machine (TBF-410, Tanita Corporation, Tokyo, Japan). The leg length of the participants will be measured from the anterior superior iliac spine to the tip of the medial malleolus by using a measuring tape. Dominant feet will be identified by asking the participants which leg is used to kick a ball [33]. All the measurements of leg length, height, body weight, and percentage of body fat will be recorded whereas body mass (BMI) will be calculated.

### Primary outcomes

#### Pain

Pain intensity will be assessed using the (VAS) [32]. It is a reliable, valid, and responsive tool, and frequently used as an outcome measure for pain [32]. It consists of a bidirectional 10 cm straight line, where 0 cm is “no pain,” and 10 cm is “worst imaginable pain,” located at either end of the line [32]. Participants will be asked to choose a number that best represents their pain level. The VAS scoring will be recorded at the baseline assessment, after every exercise session throughout the 8-week intervention, and lastly at the end of the intervention program.

#### Functional disability

The Western Ontario and McMaster Universities Arthritis Index (WOMAC) Likert version 3.1 will be used to assess functional disability [31, 34]. The WOMAC includes 24 questions. Each question will be scored using five response options ranging from 0: none, 1: mild, 2: moderate, 3: severe, to 4: extreme [31, 34]. WOMAC is designed to assess the patients’ pain perception, stiffness, and disability. The scores will be summed within the three domains, giving a total score for pain (range: 0–20), stiffness (range: 0–8), and disability (range: 0–68) [31]. Higher scores indicate greater severity across the three domains [31, 34]. The Malay version of the WOMAC (M-WOMAC) will be used in this study to better cater to the local cultural background and language spoken in the Malaysian population [35]. Based on a survey that was conducted to test the reliability and validity of the M-WOMAC among 61 women aged 45–65 years in Malaysia, the Cronbach’s alpha of 0.976 showed that the M-WOMAC was valid and reliable to be used among the local population with knee OA [35].

#### Quality of life

The Osteoarthritis Knee and Hip Quality of Life (OAKHQOL) was developed to measure the quality of life, specifically among patients with hip and knee OA [36]. The Malay version of OAKHQOL (M-OAKHQOL) will be used in the present study because its cultural and language suitability for the Malaysian population [37]. Abdul Kadir et al. [37] reported that the M-OAKHQOL is a valid and reliable instrument to assess the health-related quality of life of knee OA patients in the Malaysian population with Cronbach’s alpha values that ranged from 0.865 to 0.933. The questionnaire consists of 31 items that assess the physical activity (10 items), mental health (eight items), pain (four items), social support (three items), and social functioning (three items) as well as three additional items for relationship, sexual activity, and professional life. These three additional items are meant to be used separately [37], and they will be included in this RCT. Each of the items will be rated on a scale of 0 to 10. The final score is the mean score of all the items. All the mean scores will be added together to generate a scale of 0 (best possible QOL) to 100 (worst possible QOL) [38].

### Secondary outcomes

#### Lower limb biomechanics during gait and STS

To capture the biomechanical variable, infrared cameras with three-dimensional (3D) motion capture and analysis system will be used. Kinetic data will be collected using the force platform (Bertec, Ohio, USA) to measure the ground reaction force and joint moments whereas the kinematic data will be collected by the infrared cameras at 100Hz using the Qualisys track manager software (Qualisys, Sweden) and V3D software (version 5, C-Motion, Gothenburg, Sweden). Both the kinematic and kinetic data of the lower limb will be analyzed during the gait and STS test [39]. STS is commonly used to assess the strength and balance of the lower extremities [39]. STS has been shown to have a great intra and inter-rater reliability (ICC, 0.89) among hip and knee OA patients [40].

#### Isokinetic knee strength

Isokinetic knee flexion/extension strength will be evaluated using Biodex Dynamometer (Biodex Medical System, Shirley, NY, USA). The data to be collected will include the peak torque and muscular power of the hamstring and quadriceps. The dynamometer and chair will be adjusted to the appropriate height and length of each participant. Then, belts will be strapped on the participants to stabilize their trunk, pelvis, and contralateral thigh. The quantification of knee flexion/extension strength will be performed at the angular velocity of 180°/s [41, 42]. The speed has been chosen because it is associated with a reduction in the friction between knee cartilage, knee irritation, and other symptoms during muscle strength measurement [41]. To complete the measurement, all participants will perform each test in flexion/extension motion as hard as they can for five repetitions per set and two sets for both legs [41, 43].

### Additional measures

#### Adverse event

As a co-intervention, daily oral nonsteroidal anti-inflammatory drugs (NSAIDs) in the form of PCM 3000 mg [24, 44, 45] are provided. However, the usage of the PCM is not compulsory. Participants can consume the PCM as per their needs and record the usage in the diary provided. The pain scale (VAS) will also be recorded in the diary during the intervention period for all three groups of participants. Any adverse event during the intervention will be recorded in the provided diary. The participants will also be reminded to inform the physiotherapist immediately if they faced any problem during the eight weeks of the exercise program. The contact number is stated on the consent form. If necessary, the physiotherapist will contact the medical supervisor and co-supervisors for further investigation or management.

#### Adherence

An exercise diary will be provided to each participant from both CKC and OKC groups to ensure their adherence to the prescribed exercise at home. Similarly, participants in the control group will also be given a diary to record their daily physical activities. Additionally, a telephone call or WhatsApp message will be performed three times weekly throughout the 8-week intervention period to encourage the participants to adhere to their exercise program at home and to keep track of any adverse effects [7, 30].

### Statistical analysis

Data will be entered and analyzed using SPSS version 25 (IBM Corp., Armonk, NY, USA). Only the data from the participants who have completed at least 75% of the study intervention will be analyzed. Data from drop outs will not be included in the analysis. Descriptive data will be reported as a mean ± standard deviation. Two-way ANOVA will be used to test the within- and across- group comparison of the VAS, WOMAC, and OAKHQOL scores, and isokinetic knee muscle strength, as well as lower limb biomechanics during gait and STS. The level of significance is set at a *p*-value < 0.05. A least significant difference (LSD) post hoc test will be conducted following any significant differences from the two-way ANOVA test.

### Monitoring

The researcher will be responsible for monitoring all the subject enrollment, the reasons for dropping out, and the listing of all the adverse events to prepare all the relevant reports. The researcher will also arrange a follow-up appointment with the medical officer for any drop-out participants. Regular meetings will be held every 2 weeks between the study supervisor and researchers to monitor any adverse events from the exercise program and to ensure timely completion of participants’ recruitment. Close communication will be maintained between the assessors and researchers to monitor any problems that arise during the stages of data collection and intervention implementation. Lastly, the study supervisor will conduct monthly audits of the raw data collected, as well as the interventions in the clinical trial. Any protocol modifications that are required during the study period will be submitted to the Ethics Committee of the Universiti Sains Malaysia for approval before further implementation.

### Dissemination plans

The findings of this RCT will be presented at relevant conferences and published in academic journals. All the participants will be informed about their summary findings after the completion of the study, Furthermore, participants in the control group will be taught either OKC or CKC exercise program for their future training at home based on their preferences. All the participants will continue their follow-up with medical officers at the orthopedic clinic, hospital USM for continuity of care.

## Discussion

The prevention of knee OA is different for each individual depending on their risk factors [46]. Targeted individualized prevention techniques to ensure maximum adherence to the exercise program should be planned for each patient [46]. To achieve this, it is crucial to involved knee OA patients in the exercise program to understand their treatment preferences and improve their conditions. Apart from statistically significant differences between patient and control groups, other clinically significant differences can also be important when interpreting the trial results [47, 48]. A decrease of 2 points in the pain score and a decrease of 9.1 scores in the function subscale of the WOMAC questionnaire are examples of clinically relevant improvement [47, 49, 50]. A mere 5% change in KAM was shown to improve OA symptoms [51, 52] which will be considered as a clinically relevant improvement of the KAM in this trial. The results from this trial will provide vital evidence on clinical and biomechanical outcomes following OKC and CKC exercise programs in people with mild knee OA. Additionally, this study will help in the formulation of effective home-based exercise protocols, especially for patients with mild knee OA.

## Trial status

The protocol was registered at ClinicalTrials.gov (registration number: NCT04678609). The recruitment of participants has started on 1 August 2020 and is still ongoing. The estimated completion date of recruitment is 30 June 2022 as a result of multiple movement restriction orders in Malaysia due to the COVID-19 pandemic.

## Data Availability

Not applicable

## Abbreviations

OA: Osteoarthritis
OKC: Open kinetic chain
CKC: Closed kinetic chain
BMI: Body mass index
STS: Sit-to-stand
KAM: Knee adduction moment
RCT: Randomized controlled trial
CONSORT: CONsolidated Standards of Reporting Trials
SPIRIT: Standard Protocol Items: Recommendations for Interventional Trials
VAS: Visual analogue scale
WOMAC: Western Ontario and McMaster Universities Arthritis Index
OAKHQOL: Osteoarthritis Knee and Hip Quality of Life
HUSM: Hospital University Sains Malaysia
KL: Kellgren-Lawrence
USM: Universiti Sains Malaysia
M-WOMAC: Malay version of the WOMAC
M-OAKHQOL: Malay version of OAKHQOL
3D: Three-dimensional
5STS: Five-times-sit-to-stand test
LSD: Least significant difference
